# Vitamins Can Increase Antibiotic Effects Against Multidrug-Resistant *Pseudomonas aeruginosa* and *Acinetobacter baumannii *in an In Vitro Infection Model

**DOI:** 10.5152/eurasianjmed.2024.23145

**Published:** 2024-06-01

**Authors:** Ozgur Celebi, Demet Celebi, Sumeyye Baser, Aysegul Yilmaz, Serkan Yildirim

**Affiliations:** 1 Department of Medical Microbiology, Ataturk University Faculty of Medicine, Erzurum, Türkiye; 2 Department of Microbiology, Ataturk University Faculty of Veterinary Medicine, Erzurum, Türkiye; 3 Department of Medical Pharmacology, Amasya University Faculty of Medicine, Amasya, Türkiye; 4 Department of Pathology, Ataturk University Faculty of Veterinary Medicine, Erzurum, Türkiye

**Keywords:** *Acinetobacter baumannii*, lung cancer, *Pseudomonas aeruginosa*, synergistic effect, vitamin

## Abstract

**Background:**

Recent research has unveiled that approximately 50%-70% of patients afflicted with lung cancer also receive concurrent diagnoses of lung infections, most notably pneumonia. Among the critical multidrug-resistant pathogens frequently isolated from pneumonia-afflicted patients, *Pseudomonas aeruginosa* and *Acinetobacter baumannii* stand prominent. Here, we assessed the impact of antibiotics and vitamins on the lung cancer infection cell culture.

**Methods:**

The study delves into the antimicrobial properties of vitamins P, K, and E against *A. baumannii* and *P. aeruginosa*, employing the minimum inhibitory concentration method and scrutinizing biofilm formation within the A549 cell culture.

**Results:**

The combined application of vitamins and antibiotics exhibited a statistically significant effect on both the minimal inhibitory concentration values and biofilm formation (*P* < .05). Within the realm of fat-soluble vitamins, vitamins K and E, when integrated with antibiotics, revealed varying degrees of influence, with vitamin K displaying the most substantial fractional inhibitory concentration value. Vitamin E, on the other hand, demonstrated a comparatively weaker fractional inhibitory concentration than the other constituents. Nevertheless, it exhibited robust optical density and a pronounced capacity to inhibit biofilm formation. Moreover, among the vitamin groups, it is evident that vitamin E surpasses the efficacy of others (antibacterials affecting K < P < E). The collective application of all vitamins manifested the most potent fractional inhibitory concentration.

**Conclusion:**

The synergistic effects of vitamins with antibiotics, as evidenced in this study, may offer a promising alternative for treating multidrug-resistant *A. baumannii* and *P. aeruginosa*, subject to further investigation through molecular studies.

**Main Points**

- Hospital infections are a major problem with increasing antibiotic resistance all over the world.
- Vitamins are important in supportive treatment due to increasing antibiotic resistance.
- Vitamins act as immune modulators.

## Introduction

Individuals who cannot adequately defend against virulence factors of pathogenic microorganisms are more susceptible to infections. Immunosuppression can widely can be seen in neutropenia, transplantation, cancer, acquired immunodeficiency syndrome, extreme age (newborn, old age), trauma, burns, diabetes, alcohol addiction, liver cirrhosis, splenectomy, intravenous drug drug use (glucocorticoid, cyclosporine, etc.), and chronic kidney failure. Cancer is still the second most common cause of death and a major public health issue. When it comes to all types of cancer, lung (12.7%), breast (10.9%), stomach (7.81%), and colon (9.7%) are the most common. Lung cancer constitutes the most prevalent malignancy. It is a prominent contributor to cancer-related fatalities.^1^

Pulmonary infections are significantly effective in cancer patients.^1^ *Acinetobacter* spp., among them, is one of the most challenging infections that increases morbidity and mortality rates, especially in intensive care units. *Acinetobacter baumannii*, classified as a red alert pathogen list, is the most common cause of complications in people with different diseases, especially in cardiovascular disease and patients with cancers.^2^ *A. baumannii* is a multidrug-resistant microorganism that the World Health Organization included as a priority pathogen in 2017.^1^

An increase in lung cancer prevalence and adverse effects of chemotherapy promotes researchers to find novel medicinal agents with less toxic effects on normal cells and more selectivity, besides decreased bacterial infection incidence.^3^ Although alternative antimicrobial and vaccine studies conducted in the last 10 years are promising, the problem continues worldwide. Meropenem is a type of carbapenem with a broad antibacterial spectrum and can be effective against multidrug-resistant bacteria. Therefore, it is frequently preferred as an antibiotic for treating *A. baumannii* infections. However, the sensitivity and resistance of the bacteria to the antibiotic lead to the search for alternative treatment.^4^ Piperacillin tazobactam is a combination antibiotic consisting of beta-lactamase and piperacillin frequently used to treat complicated hospital-acquired infections.^5,6^ However, a limited number of randomized studies have compared the effectiveness of intermittent infusion with continuous infusion for this specific group of antibiotics.^7^ Polyphenols, which are prevalent in botanical species, exhibit many anticarcinogenic characteristics. These include the ability to induce apoptosis and inhibit cancer cell proliferation, inflammation, tumor growth, metastasis, and angiogenesis. Moreover, they can regulate the immune system’s reaction and safeguard healthy cells from damage caused by free radicals.^8^ Vitamin deficiency has been recognized as one of the underlying causes of pneumonia in immunocompromised patients.^9,10^ Studies have shown that alpha-tocopherol, the most beneficial vitamin E form, increases acquired immune resistance.^11^ Vitamin K deficiency is rare in elderly individuals. However, studies show that vitamin K has antimicrobial activity against *Staphylococcus aureus, Bacillus anthracis, Streptococcus agalactiae, Streptococcus pyogenes, *and* Helicobacter pylori.* ^11^ The flavonoid compound called vitamin P is isolated from vitamin C. Flavonoids are compounds with antiallergic, antioxidant, enzyme inhibitory, and antibacterial activities.^12^ Carbapenem resistance acquired through biofilms is a major problem worldwide and in our country. We searched the impact of these compound combinations on oxidative lesions within both mitochondrial and nuclear DNA in cells subjected to infection by *A. baumannii* and *Pseudomonas aeruginosa*. This investigation was conducted through the utilization of immunohistochemical analysis on suspensions derived from the study of their effects on immune-suppressive lung cells and healthy lung cells, following exposure to carefully determined doses.

## Material and Methods

### Chemicals and Reagents

Vitamins (E, K1, P), fetal calf serum, 9% isotonic sodium chloride solution, Dulbecco’s Modified Eagle’s Medium (DMEM), ethanol, tryptic soy broth, blood agar, crystal violet solution, antibiotic antimitotic solution (100×), phosphate buffer solution (PBS), agar and trypsin-EDTA (Ethylenediaminetetraasetic acid) obtained from Sigma (St. Louis, Mo, USA) were used in this study.

### Bacterial Strain Preparation

The bacterial stock of* A. baumannii *(ATCC 19606) and *P. aeruginosa* (ATCC 27853) was added to 100 µL of 2 g peptone, 200 mL: 2 g NaCl, 1 g yeast extract (Luria–Bertani) broth medium at 150 rpm at 37°C. It was produced after 24 hours of incubation.

### Minimum Inhibition Concentration Values

Minimum inhibition concentration values were ascertained for a range of concentrations encompassing vitamin E (10, 5, 2.5, 1.25, and 0.625 mg/mL), vitamin K1 (5, 2.5, 1.25, 0.625, and 0.312 mg/mL), vitamin P (10, 5, 2.5, 1.25, and 0.625 mg/mL), and meropenem (16, 8, 4, 2, 1, 0.5, and 0.25 µg/mL) against *A. baumannii* (ATCC 19606). In a 96-well format, Mueller Hinton Broth (MHB) medium was introduced, with a subsequent addition of 180 µL to each well. Subsequently, 20 µL of *A. baumannii* (ATCC 19606) suspension [10^6^ colony-forming units (CFU)/mL] was introduced. The plates were then incubated at 37°C, and after a 24-hour incubation period, a water-soluble salt solution of tetrazolium chloride (5 mg/mL), serving as a biological indicator, was incorporated into the wells. Similarly, the MIC values for vitamin E (10, 5, 2.5, 1.25, 0.625, 0.312, and 0.156 mg/mL), vitamin K1 (5, 2.5, 1.25, 0.625, and 0.312 mg/mL), vitamin P (10, 5, 2.5, 1.25, and 0.625 mg/mL), and piperacillin tazobactam (16, 8, 4, 2, 1, 0.5, and 0.25 µg/mL) were determined against *P. aeruginosa* (ATCC 27853) utilizing the microdilution method. Tryptic Soy Broth (TSB) medium was employed, with 180 µL of each dilution inoculated into 96-well plates. Subsequently, 20 µL of *P. aeruginosa* (ATCC 27853) suspension (10^6^ CFU/mL) was introduced into each well, followed by incubation at 37°C for 24 hours.^13^

### Biofilm Analysis

Biofilm assessment was conducted using the microplate methodology. *A. baumannii *and *P. aeruginosa* cultures were incubated in MHB medium at 37°C for a duration of 18-24 hours. A total of 100 µL of the tested vitamin content was added to the MHB medium in a 96-well plate. Bacterial suspensions were meticulously prepared, standardizing their density by the McFarland 0.5 standard chart. Subsequently, 100 μL of these standardized suspensions were introduced into flat-bottomed microplate wells and further incubated for 24 hours at 37°C. Following the incubation, the wells underwent thorough rinsing with distilled water, and any adherent cell residues associated with the biofilm were subsequently stained with a 1% crystal violet solution (MilliporeSigma) at 37°C for 15 minutes. Biofilms formed by the bacteria were captured through photographic documentation after the removal of excess dye via rinsing with water. For the quantitative assessment of biofilm formation, optical densities were determined utilizing an ELISA reader (Biotek ELX800; BioTek Instruments, Inc., USA) at an optical density (OD) of 450 nm, with background subtraction at OD 630 nm. Throughout the experimentation, a sterile TSB medium served as the negative control for reference purposes.^13^

### Drug and Vitamin Preparation

Preparation of Antibiotic Stock Solution: The dilution range of imipenem was determined to be 0.25-64 µg/mL.

Dilutions P, E, and K1 were prepared at 4 times the required final concentration in the well. Then, 100 µL of MHB broth was poured into each well. One hundred microliters of meropenem was diluted in half and distributed, and then 100 µL was added to the wells in which vitamins P, E, and K1 were diluted 10 mg/mL sequentially. Medium was added to the negative control well and bacteria to the positive control well. All wells received 5 µL of the antimicrobial agent except for the control wells.

### Fractional Inhibitory Concentration Index—Combination

Fractional inhibitory concentration (FIC) results were interpreted with reference to Mackay et al.^14^

Σ FIC index formula:

Synergistic; ≤0.5

Additive; >0.5 and <1

İneffective; ≥1 and 4 ≤

Antagonism; >4 was calculated as a result of the measurements.

### Cells and Cell Cultures

Cultures of A549 cells (CCL-185, ATCC) were procured for this research. To elaborate, the A549 cells were initially placed in a fresh culture medium made up of DMEM, supplemented with 10% fetal bovine serum and 1% antibiotic solution comprising penicillin, amphotericin B, and streptomycin. Subsequently, these resuspended cells were evenly distributed into Corning 24-well plates, after which they were securely housed within a controlled incubation environment (5% CO_2_, 37°C). By established procedures, the cells were resuspended as per previously documented methods. Once the cells had proliferated to approximately 85% confluence, a criterion evaluated by the McFarland 0.5 scale, a bacterial suspension was introduced into the cellular milieu. Following a 30-minute incubation period, the experimental treatments were applied and allowed to incubate for 24 hours.^15^

### MTT Assay

For MTT analysis, at the end of the time, 10 µL of MTT solution (Millipore Sigma) was added to each well plate following the commercial procedure. Following the fourth hour, 100 µL of DMSO (Millipore Sigma) was added to determine the optical density using a Multiskan™ GO microplate spectrophotometer (Paisley, UK) at 570 nm.^15^

### Immunofluorescence Analysis

Cells were incubated in paraformaldehyde for 30 minutes. Then they were kept in 3% H_2_O_2 _for 5 minutes. After dropping 0.1% Triton-X solution on them, they were rinsed in PBS and kept for 15 minutes. Then protein blocks were added and held in the dark for 5 minutes. Afterwards, the primary antibody (8-OHdG cat no: sc-66036, dilution ratio: 1/100, Paisley, UK) was added in a dropwise manner and incubated according to the manufacturer’s instructions. An immunofluorescent secondary antibody was preferred as the secondary marker (FITC Cat No: ab6785 Dilution Ratio: 1/500, Paisley, UK) for 45 minutes in darkness. Then DAPI (4′,6-diamidino-2-phenylindole) mounting medium (Cat no: D1306 Dilution Ratio: 1/200, Paisley, UK) was added and kept for 5 minutes in darkness. It was then covered with a coverslip and fluorescence microscopy was performed (Zeiss Axio, Jena, Germany).

### Statistical Analyses

The statistical definitions of the data were the mean and standard deviation (mean ± SD). The treatment groups were compared to the healthy controls using one-way analysis of variance and Tukey tests. The test determined the significance levels *P *< .05 and *P*< .001, and the results are shown as the mean ± SD.

## Results

### Microbiological Results

The effects of vitamins P, E, and K1 and meropenem on the biofilm formation of *A. baumannii* were evaluated with a colorimetric experiment in the microplate method. The minimum inhibitory concentrations of the antimicrobial agents used for *A. baumannii* are as follows: meropenem third well/2 mg/mL, vitamin P 3. well/1.25 mg/mL, vitamin K1 seventh well/0.078 mg/mL, vitamin E fourth well/0.625 mg/mL, for *P. aeruginosa*; piperacillin tazobactam fourth well/3 mg/L, vitamin P fourth well/0.625 mg/mL, vitamin K1 fourth well/0.625 mg/mL, vitamin E sixth well/0.156 mg/mL.

Considering the FIC dose ranges used in the treatment of infection due to *P. aeruginosa *, vitamin K1 + piperacillin tazobactam, vitamin P + piperacillin tazobactam, vitamin E + piperacillin tazobactam 5 mg/mL + 0.5 µg/mL, and K1 + P + E + piperacillin tazobactam 2.5 mg/mL + 2.5 mg/mL + 5 mg/mL + 4 mg/mL. Vitamin K, vitamin E, and K1 + P + E showed a synergistic effect in the combination of antibiotics, while the combination of vitamin P and antibiotics showed an additive effect. In addition, OD values of P, K1, E, and P + K1 + E antibiotic combinations were determined as 1.032, 1.423, 1.112, and 1.347, respectively, in the presence of biofilm under 570 nm.

Considering the FIC dose ranges used in the treatment of infection due to *A. baumannii*, vitamin K1 with meropenem was important in terms of its inhibition value, the antimicrobial effect of vitamin E was not observed. The greatest support for the destruction of the biofilm layer was seen in the combination of vitamin K1 + meropenem. There was no noticeable difference in the combined effect of all combinations compared to the other groups. *A. baumannii* and *P. aeruginosa* for in the evaluation, the strongest FIC concentration was found at K1 + P + E and antimicrobial 2.5 mg/mL + 2.5 mg/mL + 0.5 µg/mL doses. These findings are shown in Table 1 and 2.

### MTT Assay

**Figure 1.**
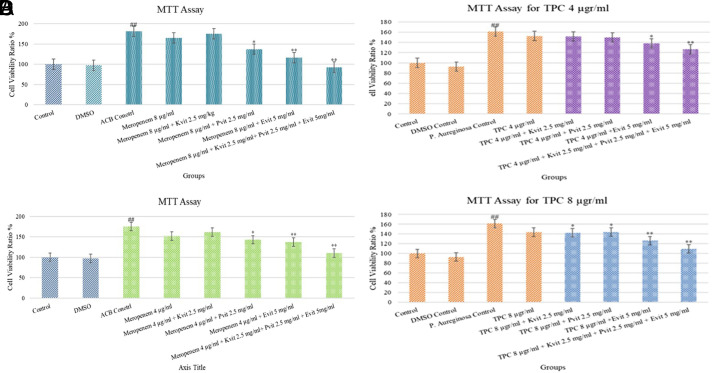
A, B, C, and D show cell viability of A549 cells 24 hours. Figure 1A and Figure 1C show meropenem effects. The meropenem experiment groups consist of control, DMSO control, ACB (* Acinetobacter baumannii*) Control, meropenem 4 µg/mL, meropenem 4 µg/mL + K vitamin 2.5 mg/mL, meropenem 4 µg/mL + P vitamin 2.5 mg/mL, meropenem 4 µg/mL + E vitamin 5 mg/mL and meropenem 4 µg/mL + K vitamin 2.5 mg/mL + P vitamin 2.5 mg/mL + E vitamin 5 mg/mL. Figure 1B and 1D show TPC (piperacillin tazobactam) effects. The TPC experiment group consists of control, DMSO control, P. *aeruginosa* control, TPC 8 µg/mL, TPC 8 µg/mL + K vitamin 2.5 mg/mL, TPC 8 µg/mL + P vitamin 2.5 mg/mL, TPC 8 µg/mL + E vitamin 5 mg/mL, and TPC 8 µg/mL + K vitamin 2.5 mg/mL + P vitamin 2.5 mg/mL + E vitamin 5 mg/mL. The significant difference was determined (control group and bacterial control group ^#^ *P* < .05, ^##^ *P* < .001) (control group and treatment *P < .05, **P < .001).

The cytotoxic effect of meropenem (MER) and vitamins was determined using the MTT method at the end of 24 hours (Figure 1). The control and ABC control were compared. Meropenem 4 and 8 µg/mL, and vitamins were compared with the ABC control group. The cell viability ratio of the control group was 100%. Statistically, the ABC control group was different from the control group (*P *< .001P). The viability ratio of the treatment groups was decreased by vitamins (in both MER 4 and µg/mL), especially at the E and P doses (<.001P). In the 4 µg/mL MER combination groups, the lowest viability occurred in the 3 combination groups (viability ratio was 110.05%) (<.001P). The 8 µg/mL MER combination groups showed more toxicity at the same vitamin doses. In the 8 µg/mL MER combination groups, the lowest viability was in the 3 vitamin combination groups (viability ratio was 92.86%) (<.001). Between the vitamin groups, it is evident that the E vitamin is more effective than the others (antibacterials affect K1 < P < E vit).

The cell viability rate of the treatment was compared to *P. aeruginosa *. There were no significant differences between piperacillin tazobactam 4 µg/mL, piperacillin tazobactam 4 µg/mL + K1 vitamin 2.5 mg/kg, piperacillin tazobactam 4 µg/mL + P vitamin 2.5 mg/mL, and *P. aeruginosa* group. A significant difference was seen in piperacillin tazobactam 4 µg/mL + E vitamin 5 mg/mL (*P *< .05) and piperacillin tazobactam 4 µg/mL + K1 vitamin 2.5 mg/mL + P vitamin 2.5 mg/mL + E vitamin 5 mg/mL (*P *< .001) respectively. Figure 1 result shows an increase in piperacillin tazobactam dose to 8 µg/mL increases antibacterial effects, and also vitamin combination acts more effectively. Significant differences were detected between piperacillin tazobactam 8 µg/mL, piperacillin tazobactam 8 µg/mL + K1 vitamin 2.5 mg/kg, piperacillin tazobactam 8 µg/mL + P vitamin 2.5 mg/mL treatment (*P *< .05) and *Pseudomonas* group. The *P *< .001 significant differences were seen in piperacillin tazobactam 8 µg/mL + E vitamin 5 mg/mL and piperacillin tazobactam 8 µg/mL + K1 vitamin 2.5 mg/mL + P vitamin 2.5 mg/mL + E vitamin 5 mg/mL.

### Immunofluorescence Staining Results

**Figure 2.**
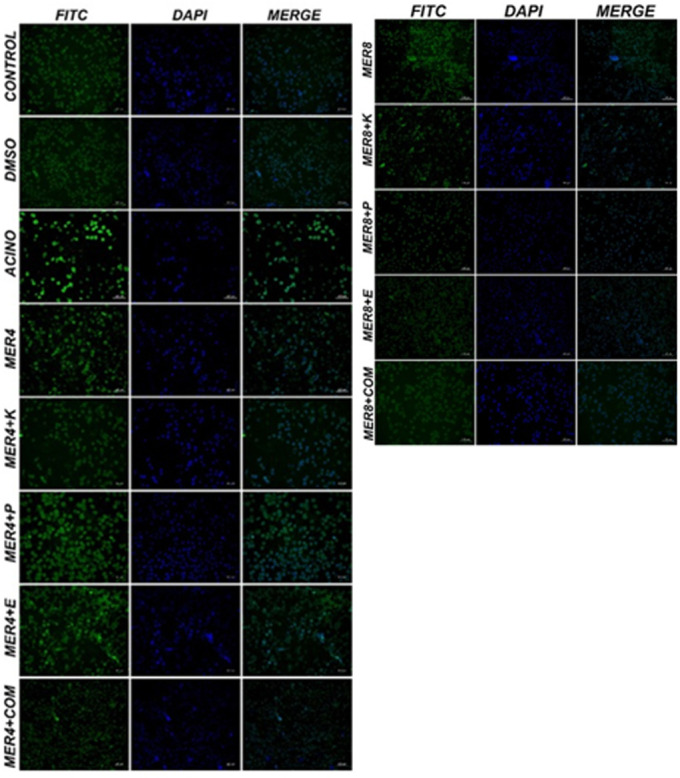
Cell culture, 8-OHdG expression, immunofluorescence, Bar: 100 µm.

*A. baumannii* for the control group, 8-OHdG expression was evaluated as negative in the examination performed on the A549 cell line (Figure 2 and Table 3). DMSO group, 8-OHdG expression was evaluated as negative in the examination performed on the A549 cell line. In the Acinetobacter group, upon examination of the A549 cell line, severe cytoplasmic 8-OHdG expression was detected in A549 cells. In the meropenem group, mild 8-OHdG expression was observed in the A549 cells in the examination performed on the A549 cell line. MER 4 µg/mL + vitamin showed very mild 8-OHdG expression. MER 4 µg/mL + E vitamin +P vitamin + K vitamin showed a low expression level. In contrast, the 8 µg/mL MER result shows a more prominent decrease in the 8-OHdG expression level than the 4 µg/mL MER expression levels. The lowest expression of 8-OHdG was observed in the 8 µg/mL MER + E vitamin +P vitamin + K vitamin combination group (Figure 2).

**Figure 3.**
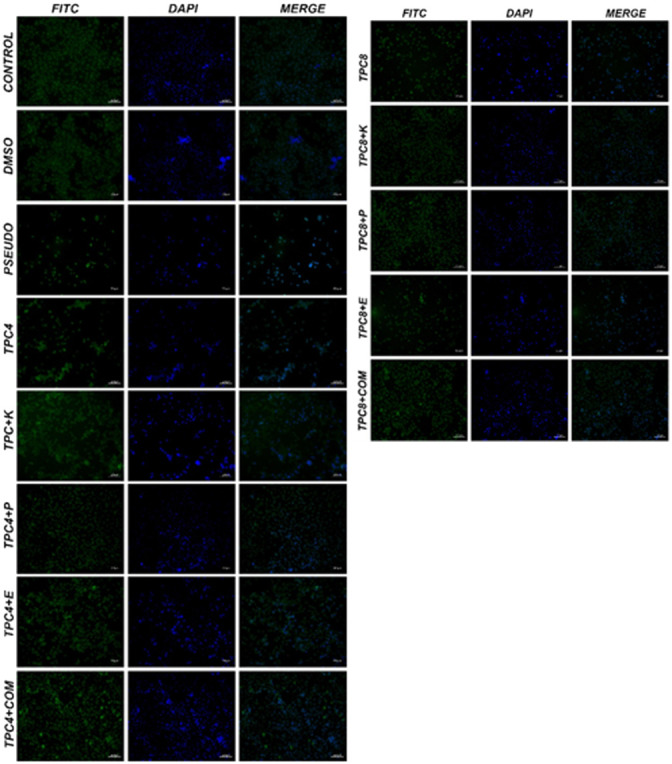
Cell culture, intracytoplasmic 8-OHdG expressions in cells (FITC), IF, Bar: 100µm.

*Pseudomonas aeruginosa* for control and DMSO group, 8-OHdG expressions in cells were evaluated as negative. The *Pseudomonas* group exhibited very intense intracytoplasmic 8-OHdG expression. The TPC4 group represented intense cytoplasmic 8-OHdG expression. Moderate 8-OHdG expression was detected in the TPC4+K group. Moderate intracytoplasmic 8-OHdG expression was found in the TPC4+P group. The TPC4+E group demonstrated moderate cytoplasmic 8-OHdG expression. The TPC4+COM group revealed mild cytoplasmic 8-OHdG expression. A significant difference was noted when compared with the *Pseudomonas* group (*P* < .05). In the TPC8 group, moderate cytoplasmic 8-OHdG expression was discovered. TPC8+K group suggested mild intracytoplasmic 8-OHdG expression. The TPC8+P group indicated mild 8-OHdG expression. TPC8+E group exhibited mild intracytoplasmic 8-OHdG expression. In TPC8+COM group very mild intracytoplasmic 8-OHdG expression occurred. There was a significant difference when compared with the *Pseudomonas* group (*P* < .05). Table 4 and Figure 3 illustrate the findings of the statistical studies of the immunofluorescent staining.

## Discussion

Depending on the severity of the course of lung cancer and the region of residence, it is responsible for 20% of the mortality rate. In cancer-related complications, deaths based on infection are in second place.^16^ The pathogens responsible for this condition are known as multidrug-resistant pathogens. Examples include *S. aureus*, specifically vancomycin-resistant *S. aureus*, as well as methicillin-resistant *S. aureus* (MRSA), or vancomycin-intermediate *Staphylococcus aureus*. Additionally, there are enterococci resistant to vancomycin, referred to as vancomycin-resistant enterococci or oxazolidinone-resistant enterococci. *Streptococcus pneumoniae* is another pathogen resistant to third-generation cephalosporins and penicillin, known as penicillin-resistant *Streptococcus pneumoniae*. Last, the Enterobacteriaceae family also includes strains that exhibit resistance to various antibiotics.^17^ *P. aeruginosa *, being a multifaceted opportunistic pathogen, can induce acute and chronic infections. The pathogenic nature of *P. aeruginosa* can be attributed to the presence of antibiotic resistance markers and sizable virulence factors within its genome. This, coupled with its exceptional metabolic adaptability and capability to evade numerous conditions, including the host’s immune response, has complicated the development of effective treatments and vaccines. *A. baumannii* for our study determined safe dose ranges of vitamins P, E, and K1. Biofilm effects were examined. The effective dose of the antibiotic prevented biofilm formation. In our study, the effect on biofilm formation was strong in combination with meropenem and vitamin E. In our study, the effect of vitamin K on biofilm formation was found to be significant, but it is not more effective than P and E vitamins. Vitamin P is a flavonoid group compound produced from vitamin C. Vitamin P combined with meropenem increases antibacterial ability not only by affecting FIC and MIC but also by affecting biofilm formation.

Here, we determined the antimicrobial efficacy of K1, E, P, and K1 + E + P + antibiotic combinations as an alternative treatment against *P. aeruginosa* that developed antibiotic resistance. In our study, vitamin K1 + piperacillin tazobactam, vitamin P + piperacillin tazobactam, and vitamin E + piperacillin tazobactam 5 mg/mL + 0.5 µg/mL were used in the treatment of *P. aeruginosa* infections, K1 + P + E + piperacillin tazobactam at concentrations of 2.5 mg/mL + 5 mg/mL + 0.5 µg/mL were determined. Vitamin K1, vitamin E, and K1 + P + E + showed a synergistic effect in the combination of antibiotics, while the combination of vitamin P and antibiotics showed an additive effect. In addition, OD values of K1, E, P, and K1 + P + E antibiotic combinations were determined as 1.423, 1.112, 1.032, and 1.347, respectively, in the presence of biofilm at 570 nm. In a similar study, Shahzad et al^18^ revealed that vitamins B2, B1, and B12 performed great synergistic effect with linezolid against MRSA. Vitamin B1 had a better synergy with tetracycline, rifampicin, oxacillin, and linezolid against MRSA. Fat-soluble vitamins K1 and E demonstrated good synergism against gram-negative *A. baumannii*. On the other hand, water-soluble vitamins B12, B2, and B1 were efficacious against MRSA but ineffective against *A. baumannii.* ^18^ In a study examining the antibacterial effect of vitamin E against *Escherichia coli, Pseudomonas* spp., and *S. aureus* pathogens were effective.^19^ Vitamin E has high antioxidant activity and can influence bacterial growth and prevent infection by supporting the host’s immune system.^20^ In a study, it was reported in a study that vitamin K1 has strong inhibition properties, especially against gram-negative bacteria, and even a synergistic effect was observed when combined with antibiotics.^21^ The lipophilic structure of vitamin K1, aimed at disrupting the integrity of the bacterial cell membrane, prevents pathogens from causing infection.^21^ Vitamin P or naringenin is a vitamin with an antioxidant structure, especially isolated from vitamin C.^22^ Like every vitamin with antioxidant properties, it creates a stress factor for the growth of bacteria and also supports the mechanisms as an immune modülatör.^12^ At the same time, there are studies that have effects on biofilm formation are available in the literature.^13^ In our study, it had an additive effect on FIC concentration. It has also been shown in our results that it affects biofilm formation.

This study is an in vitro application of a vitamin-antibiotic combination applicable in the treatment of *A. baumannii* and *P. aeruginosa*. As a deficiency in the study, no molecular level investigation was performed. This study should be supported by molecular studies. Efficacy should be determined in advanced studies. With these preliminary data, antibiotic–vitamin combinations should be applied to experimental animals.

Combinations of all vitamins together exhibited the most potent FIC. With molecular studies, this synergistic effect of vitamins with antibiotics can be a viable alternative in treating multidrug-resistant *A. baumannii *and* P. aeruginosa.*

## Figures and Tables

**Table 1.** Results of the Microdilution Assay with Biofilm and Minimal Inhibitory Concentration of Agents

Agent	Biofilm Optical Density Highest value	Positive Control	Negative Control	Minimum Inhibitory Concentration/Dose
Meropenem*	0.186	0.133	0.071	Third well/2 mg/mL
P vitamin*	0.518	0.536	0,142	Third well/1.25 mg/mL
K1 vitamin*	0.522	0.653	0.32	Seventh well/0.078 mg/mL
E vitamin*	0.612	1.067	0.411	Fourth well/0.625 mg/mL
Piperacillin tazobactam**	0.193	0.126	0.082	Fourth well/3 mg/L
P vitamin**	0.904	1.15	0.347	Fourth well/0.625 mg/mL
K1 vitamin**	0.601	0.757	0.425	Fourth well/0.625 mg/mL
E vitamin**	1.023	1.236	0.47	Sixth well/0.156 mg/mL

*Acinetobacter baumannii

**Pseudomonas aeruginosa.

**Table 2.** Results of the Checkerboard Assay with Biofilm and Fractional Inhibitory Concentration Indices of Agent Combinations.

Bacteria Strains, ATCC No.	Agent	Biofilm’s Highest OD Value	Positive Control	Negative Control	Fractional Inhibitory Concentration	Dose
*Acinetobacter baumannii* ATCC 19606	Vitamin K1– meropenem	1.397	0.355	0.146	0.26	0.5 µg/mL + 2.5 mg/mL
	Vitamin P– meropenem	1.167	1.156	0.574	0.30	0.5 µg/mL + 2.5 mg/mL
	Vitamin E– meropenem	1.355	0.962	0.597	0.50	0.5 µg/mL + 5 mg/mL
	Vitamins K1 + P + E– meropenem	1.195	0.978	0.554	0.25	0.5 µg/mL+ 2.5 mg/mL + 2.5 mg/mL + 5 mg/mL
*Pseudomonas aeruginosa ATCC 27853*	Vitamin K1–piperacillin/ tazobactam	1.423	0.995	0.247	0.47	2.5 mg/mL+ 0.5 µg/mL
	Vitamin P–piperacillin/tazobactam	1.032	0.967	0.379	0.60	2.5 mg/mL + 0.5 µg/mL
	Vitamin E–piperacillin/tazobactam	1.112	0.820	0.216	0.61	5 mg/mL + 0.5 µg/mL
	Vitamins K1 + P + E–piperacillin/tazobactam	1.347	1.1	0.535	0.31	2.5 mg/mL + 2.5 mg/mL + 5 mg/mL + 5 µg/mL

**Table 3.** Analysis Data and Statistical Analysis of Immunofluorescence Staining Results

Group	8-OHdG
Control	35.58 ± 1.38
DMSO	37.11 ± 3.36
ACİNO	18.31 ± 3.11**
MER4	42.32 ± 1.59^#^
MER4+K	40.72 ± 1.38^#^
MER4+P	47.35 ± 3.36^#^
MER4+E	52.41 ± 3.11^##^
MER4+COM	62.42 ± 1.59^##^
MER8	15.24 ± 1.38**
MER8+K	40.16 ± 3.36^#^
MER8+P	58.76 ± 3.11^##^
MER8+E	75.39 ± 1.59^##^
MER8+COM	95.52 ± 1.38^##^

Different symbols in the same column represent significant differences (*P *< .05 AP).

**Table 4.** Analysis Data and Statistical Analysis of Immunofluorescence Staining Results

Group	8-OHdG
Control	32.34 ± 3.34
DMSO	33.25 ± 2.6
PSEUDO	18.31 ± 3.11**
TPC4	35.24 ± 2.69^#^
TPC4+K	36.28 ± 3.13^#^
TPC4+P	37.24 ± 2.35^#^
TPC4+E	42.54 ± 4.01^#^
TPC4+COM	62.42 ± 5.38^##^
TPC8	17.24 ± 1.14**
TPC8+K	36.16 ± 3.36^#^
TPC8+P	39.76 ± 3.11^#^
TPC8+E	52.27 ± 4.12^##^
TPC8+COM	75.52 ± 6.25^##^

Different symbols in the same column represent statistical differences (*P* < .05 AP).
